# Optic chiasm involvement in multiple sclerosis, aquaporin-4 antibody-positive neuromyelitis optica spectrum disorder and myelin oligodendrocyte glycoprotein–associated disease

**DOI:** 10.1177/13524585241240420

**Published:** 2024-04-22

**Authors:** Alessia Bianchi, Rosa Cortese, Ferran Prados, Carmen Tur, Baris Kanber, Marios C Yiannakas, Rebecca Samson, Floriana De Angelis, Lise Magnollay, Anu Jacob, Wallace Brownlee, Anand Trip, Richard Nicholas, Yael Hacohen, Frederik Barkhof, Olga Ciccarelli, Ahmed T Toosy

**Affiliations:** Queen Square MS Centre, Department of Neuroinflammation, UCL Queen Square Institute of Neurology, Faculty of Brain Sciences, University College London, London, UK; Department of Medicine, Surgery and Neuroscience, University of Siena, Siena, Italy; Queen Square MS Centre, Department of Neuroinflammation, UCL Queen Square Institute of Neurology, Faculty of Brain Sciences, University College London, London, UK; Department of Medicine, Surgery and Neuroscience, University of Siena, Siena, Italy; Queen Square MS Centre, Department of Neuroinflammation, UCL Queen Square Institute of Neurology, Faculty of Brain Sciences, University College London, London, UK; Centre for Medical Image Computing, Medical Physics and Biomedical Engineering, University College London, London, UK; eHealth Centre, Universitat Oberta de Catalunya, Barcelona, Spain; Queen Square MS Centre, Department of Neuroinflammation, UCL Queen Square Institute of Neurology, Faculty of Brain Sciences, University College London, London, UK; MS Centre of Catalonia (Cemcat), Vall d’Hebron Institute of Research, Vall d’Hebron Barcelona Hospital Campus, Barcelona, Spain; Queen Square MS Centre, Department of Neuroinflammation, UCL Queen Square Institute of Neurology, Faculty of Brain Sciences, University College London, London, UK; Centre for Medical Image Computing, Medical Physics and Biomedical Engineering, University College London, London, UK; Queen Square MS Centre, Department of Neuroinflammation, UCL Queen Square Institute of Neurology, Faculty of Brain Sciences, University College London, London, UK; Queen Square MS Centre, Department of Neuroinflammation, UCL Queen Square Institute of Neurology, Faculty of Brain Sciences, University College London, London, UK; Queen Square MS Centre, Department of Neuroinflammation, UCL Queen Square Institute of Neurology, Faculty of Brain Sciences, University College London, London, UK; Queen Square MS Centre, Department of Neuroinflammation, UCL Queen Square Institute of Neurology, Faculty of Brain Sciences, University College London, London, UK; Department of Neurology, The Walton Centre NHS Foundation Trust, Liverpool, UK; Department of Neurology, Cleveland Clinic Abu Dhabi, Abu Dhabi, United Arab Emirates; Queen Square MS Centre, Department of Neuroinflammation, UCL Queen Square Institute of Neurology, Faculty of Brain Sciences, University College London, London, UK; Biomedical Research Centre, National Institute for Health Research (NIHR), University College London Hospitals (UCLH), London, UK; Queen Square MS Centre, Department of Neuroinflammation, UCL Queen Square Institute of Neurology, Faculty of Brain Sciences, University College London, London, UK; Biomedical Research Centre, National Institute for Health Research (NIHR), University College London Hospitals (UCLH), London, UK; Division of Brain Sciences, Department of Medicine, Imperial College London, London, UK; Queen Square MS Centre, Department of Neuroinflammation, UCL Queen Square Institute of Neurology, Faculty of Brain Sciences, University College London, London, UK; Department of Neurology, Great Ormond Street Hospital For Children NHS Foundation Trust, London, UK; Queen Square MS Centre, Department of Neuroinflammation, UCL Queen Square Institute of Neurology, Faculty of Brain Sciences, University College London, London, UK; Centre for Medical Image Computing, Medical Physics and Biomedical Engineering, University College London, London, UK; Biomedical Research Centre, National Institute for Health Research (NIHR), University College London Hospitals (UCLH), London, UK; Department of Radiology and Nuclear Medicine, Amsterdam UMC, Vrije Universiteit Amsterdam, Amsterdam, The Netherlands; Queen Square MS Centre, Department of Neuroinflammation, UCL Queen Square Institute of Neurology, Faculty of Brain Sciences, University College London, London, UK; Biomedical Research Centre, National Institute for Health Research (NIHR), University College London Hospitals (UCLH), London, UK; Queen Square MS Centre, Department of Neuroinflammation, UCL Queen Square Institute of Neurology, Faculty of Brain Sciences, University College London, London, UK

**Keywords:** Optic chiasm, optic neuritis, multiple sclerosis, aquaporin4-antibody neuromyelitis optica spectrum disorder, myelin oligodendrocyte glycoprotein antibody–associated disease, magnetisation transfer ratio

## Abstract

**Background:**

Optic neuritis (ON) is a common feature of inflammatory demyelinating diseases (IDDs) such as multiple sclerosis (MS), aquaporin 4-antibody neuromyelitis optica spectrum disorder (AQP4 + NMOSD) and myelin oligodendrocyte glycoprotein antibody–associated disease (MOGAD). However, the involvement of the optic chiasm (OC) in IDD has not been fully investigated.

**Aims:**

To examine OC differences in non-acute IDD patients with (ON+) and without ON (ON−) using magnetisation transfer ratio (MTR), to compare differences between MS, AQP4 + NMOSD and MOGAD and understand their associations with other neuro-ophthalmological markers.

**Methods:**

Twenty-eight relapsing-remitting multiple sclerosis (RRMS), 24 AQP4 + NMOSD, 28 MOGAD patients and 32 healthy controls (HCs) underwent clinical evaluation, MRI and optical coherence tomography (OCT) scan. Multivariable linear regression models were applied.

**Results:**

ON + IDD patients showed lower OC MTR than HCs (28.87 ± 4.58 vs 31.65 ± 4.93; *p* = 0.004). When compared with HCs, lower OC MTR was found in ON + AQP4 + NMOSD (28.55 ± 4.18 vs 31.65 ± 4.93; *p* = 0.020) and MOGAD (28.73 ± 4.99 vs 31.65 ± 4.93; *p* = 0.007) and in ON− AQP4 + NMOSD (28.37 ± 7.27 vs 31.65 ± 4.93; *p* = 0.035). ON+ RRMS had lower MTR than ON− RRMS (28.87 ± 4.58 vs 30.99 ± 4.76; *p* = 0.038). Lower OC MTR was associated with higher number of ON (regression coefficient (RC) = −1.15, 95% confidence interval (CI) = −1.819 to −0.490, *p* = 0.001), worse visual acuity (RC = −0.026, 95% CI = −0.041 to −0.011, *p* = 0.001) and lower peripapillary retinal nerve fibre layer (pRNFL) thickness (RC = 1.129, 95% CI = 0.199 to 2.059, *p* = 0.018) when considering the whole IDD group.

**Conclusion:**

OC microstructural damage indicates prior ON in IDD and is linked to reduced vision and thinner pRNFL.

## Introduction

Inflammatory demyelinating diseases (IDDs) represent a spectrum of heterogeneous disorders affecting the central nervous system. Multiple sclerosis (MS), aquaporin 4-antibody neuromyelitis optica spectrum disorder (AQP4 + NMOSD) and myelin oligodendrocyte glycoprotein antibody–associated disease (MOGAD) are the most defined forms.^1^

Optic neuritis (ON) is an acute, inflammatory condition primarily involving the optic nerve and is frequently observed in IDD, although with different patterns.^2–5^ In MS, ON is often unilateral, characterised by short lesions and tends to recover well.^6,7^ In contrast, AQP4 + NMOSD-associated ON can be unilateral or bilateral, more frequently associated with severe visual loss and limited recovery if untreated. AQP4 + NMOSD optic nerve lesions are extensive, involving more than half of its length and posteriorly located. In MOGAD, ON is more frequently bilateral and is associated with severe visual loss, yet there is potential for favourable clinical recovery.^8^ Optic nerve lesions in MOGAD patients are often long and anteriorly located, leading to the frequent observation of optic disc oedema in acute ON. In addition, perineural enhancement has been documented in some cases.^6,9^

Magnetisation transfer (MT) is an advanced magnetic resonance imaging (MRI) technique used to assess the exchange of proton magnetisation between tissue macromolecules and mobile water molecules, a phenomenon typically quantified by the magnetisation transfer ratio (MTR).^10^ While MTR has been associated with myelin content, MTR changes may also reflect neuroaxonal loss.^11^ In the optic nerve, MTR has proven to be a valuable measure of early demyelination and predictor of axonal loss and remyelination after acute ON.^12,13^ We recently reported that MTR values can also help the discrimination between relapsing-remitting multiple sclerosis (RRMS) and AQP4 + NMOSD.^14^

The optic chiasm (OC) may be pathologically altered with acute ON through direct lesional involvement, which is more frequently observed in AQP4 + NMOSD,^2,15^ or from secondary post-acute neurodegeneration from more anterior optic nerve lesions.^16–18^ These chiasmatic alterations can be related to visual impairment.^9,19^ However, the comprehensive quantification of OC involvement and its impact on visual outcomes in the context of these three IDDs has not been fully explored. We conducted a prospective study on a cohort of non-acute IDD patients both with (ON+) and without (ON−) previous ON to assess (1) microstructural OC MTR changes between ON+ and ON− patients; (2) whether the degree of the OC MTR changes differs between RRMS, AQP4 + NMOSD and MOGAD and (3) and if OC MTR is associated with residual visual/ophthalmic outcomes in these patients.

## Patients and methods

From a previously described cohort,^14^ 80 patients and 32 healthy controls (HCs) were selected. Inclusion criteria for patients were (1) diagnosis of RRMS according to 2017 revised criteria^20^ or AQP4 + NMOSD according to 2015 Wingerchuk’s criteria^21^ or MOGAD, defined as MOG-Ab positivity in the context of an acute demyelinating event in patients presenting with a MOGAD phenotype previously described;^22^ (2) no clinical relapses in the previous 6 months; (3) no ophthalmic conditions; (4) age above 18 years at the time of assessment and (5) no major contraindications to MRI. The study was conducted in accordance with the International Conference on Harmonisation guidelines for Good Clinical Practice and the Declaration of Helsinki. All participants gave informed consent upon admission to the study, which was approved by the National Research Ethics Service (NRES) Committee London Bloomsbury.

All patients underwent detailed clinical evaluation, peripapillary retinal nerve fibre layer (pRNFL) and macular ganglion cell–inner plexiform layer (GCIPL) thickness measurements with optical coherence tomography (OCT) scanning and MRI. Episodes of ON were identified by clinicians through clinical information collected from medical records. ON was defined as subacute, monocular visual loss associated with pain during eye movement, with objective evidence of an optic neuropathy (e.g. impaired best-corrected visual acuity, dyschromatopsia, relative afferent pupillary defect and/or optic disc pallor/swelling).^2,23^ The number of separate inflammatory events was determined for each eye of each patient. Visual assessment was performed for each eye separately with high contrast letter visual acuity using the retro-illuminated Early Treatment Diabetic Retinopathy Study chart at 4 m with best correction. Higher logMAR scores reflect worse visual acuity; a score of 1.7 was assigned when no letters could be correctly identified by the patient. To test the association between MTR value in the OC and the clinical outcome, both (1) visual acuity as an average between the two eyes and (2) visual acuity in the worst eye were entered in separate analyses.

Patients and controls underwent pRNFL and GCIPL OCT scanning using Heidelberg Eye Explore 1.10.2.0 (Spectralis version 6.9a, Heidelberg Engineering, Heidelberg, Germany). Optic nerve thicknesses at 3.4 mm ring scan were extracted. A quality check was performed according to the international OSCAR-IB criteria.^24^

All participants underwent MRI using a 3T Achieva system (Philips Medical Systems, Best, The Netherlands) and 32-channel head coil based at the NMR research unit, Queen Square, London. Left and right optic nerves were acquired separately with (1) coronal-oblique two-dimensional (2D) fat-suppressed turbo spin-echo T2-weighted (T2w) imaging with coronal-oblique plane orthogonal to the long axis of the optic nerve (number of slices 20, slice thickness 3 mm, no slice gap, field-of-view (FOV) 160 × 160 × 160 mm^3^, voxel size 0.5 × 0.5 mm^2^) and (2) Magnetisation Transfer imaging (MTI), using identical scan geometry as above (number of slices 20, FOV 160 × 160 × 60 mm^3^, acquisition voxel size 0.75 × 0.75 × 3 mm^3^, reconstruction voxel 0.5 × 0.5 × 3 mm^3^). MTI comprises of a three-dimensional (3D) slab-selective fast field-echo sequence with two echoes, performed with and without Sinc–Gaussian-shaped MT saturating pulses (MTon and MToff, respectively) of nominal angle α = 360°, offset frequency 1 kHz, duration 16 ms. Participants were asked to close their eyes during scanning. Two sets of MT images were acquired for each eye to improve signal-to-noise and were pre-processed to generate MTon average and MToff average and then co-registered to native T2w space.

**Figure 1.**
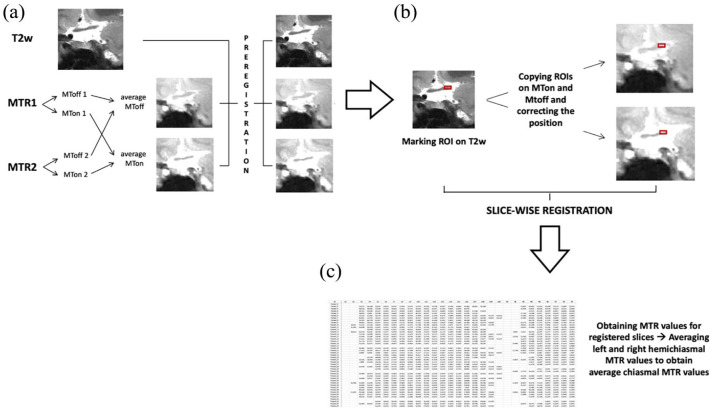
The figure illustrates the processing pipeline for the magnetisation transfer ratio (MTR) optic pathway analysis: (a) Two MTR acquisitions (2 echo-times) were obtained for each eye and they were pre-processed to generate two MTon and two MToff images. From these two different echo time MT images, average MToff and average MTon images were created, which were subsequently pre-registered to the native T2w image, (b) regions of interest (ROIs) were manually delineated slice-by-slice to cover the right and left section of the optic chiasm independently on each T2w acquisition. Afterwards, these ROIs were transposed onto the pre-registered MTon and MToff average images and manually adapted to delineate the same area on the pre-registered MTon and MToff average. Subsequently, to correct for small nerve motion, the three ROIs’ centre-of-mass slice-wise were aligned to a common space (T2w and registered average MTon and MToff) and (c) MTR values for both left and right optic hemichiasms were obtained from the registered images. For each patient, right and left MTR values were averaged to obtain a single averaged chiasmal MTR value that was then impute into the statistical analysis.

The average number of slices for the OC was 1.73 slices (median = 1, range = 1–2). Chiasmal MTR processing is described in Figure 1. Manually delineated Regions of Interest (ROIs) were created slice-by-slice to encompass the right and left sections of the OC (optic hemichiasms) independently on each T2w acquisition. OC ROIs were delineated on the T2w coronal orbital MRI scans by two experienced raters (R.C. and A.B.) for left and right optic nerve acquisitions using JIM 6.0 (Xinapse systems, http://www.xinapse.com). Raters were blinded to diagnosis during region of interest (ROI) delineation. There was a high degree of consistency in inter-rater agreement in identifying the optic nerve ROIs, as indicated by a 95% Cohen’s kappa coefficient. ROIs were subsequently transferred from T2w images to the pre-registered MTon and MToff average images and manually adjusted to ensure consistent delineation of the same area on T2w, MTon and MToff images. To additionally account for minor nerve motion, the centre-of-mass of the three ROIs was aligned slice-wise to a common space built on T2w and registered average MTon and MToff images. The registered images were then utilised to extract MTR values for both the left and right optic hemichiasms. Finally, the MTR values from the hemichiasms were statistically averaged to obtain a single chiasmal MTR value.

## Statistical analysis

Statistical analysis was performed using STATA/MP software version 17.0 (Copyright 1985-2021 StataCorp LLC).

To assess associations between chiasmal MTR values and ON, we categorised subjects in three different ways: (1) ON− IDD (*n* = 32) vs ON + IDD (*n* = 48) vs HCs (*n* = 32); (2) ON+ RRMS (*n* = 11) vs ON+ AQP4 + NMOSD (*n* = 15) vs ON + MOGAD (*n* = 22) vs HCs (*n* = 32) and (3) ON IDD (*n* = 32) vs IDD with one previous ON (*n* = 22) vs IDD with two previous ON (*n* = 10) vs IDD with three or more previous ON (*n* = 16) vs HCs (*n* = 32).

Clinical and demographic characteristics of groups were compared using *t*-tests or analysis of variance (ANOVA) for continuous variables and Chi-square test for independence to compare the groups in categorical variables.

Multivariable linear regression models were fitted to estimate the associations between OC MTR as the continuous response variable and different clinical categorical predictors described above,^25^ adjusting for potential confounders, including age, sex, disease duration and MRI scanner software upgrade which occurred during the study.

We also tested associations between (1) OC MTR versus visual acuity and (2) OC MTR versus pRNFL and GCIPL independently. Linear regression models were fitted with logMAR acuity, pRNFL thickness or GCIPL thickness as outcomes in two ways: (1) average logMAR and average pRNFL/GCIPL across both eyes or (2) worse logMAR and thinner pRNFL/GCIPL between both eyes for each individual. OC MTR and other potential confounders were entered as independent variables.

All inferences used a type I error rate of *p* < 0.05 for clear statistical significance.

## Results

### Participant characteristics

Eighty patients with IDD, including 28 RRMS (18 females, mean (±SD) age: 45.5 (±11.8) years), 24 AQP4 + NMOSD (18 females, mean age: 49.9 (±12.7) years) and 28 MOGAD (19 females, mean age: 36.4 (±17.1) years) patients and 32 HCs were enrolled in the study. IDD patients were older than HCs (43.6 years ± 15.0 vs 35.2 years ± 12.4; *p* = 0.007), while no significant differences were found for gender (females: 68.75% vs 68.75%). The age and gender distributions were similar between ON+ IDD and ON− IDD patients. Demographic characteristics of the different groups are summarised in Tables 1 and 2.

**Table 1.** Clinical characteristics of the population of study.

Demographic characteristics	RRMS (*n* = 28)	AQP4 + NMOSD (*n* = 24)	MOGAD (*n* = 28)	HCs (*n* = 32)	*p*
Gender, M / F (male %)	10 / 18 (35.7%)	6 / 18 (25.0%)	9 / 19 (32.1%)	10 / 22 (31.3%)	*p* = 0.872^b^
Age, years (mean ± SD)	45.5 ± 11.8	49.9 ± 12.7	36.4 ± 17.1	35.2 ± 12.4	*p* = 0.002^a^
Age at disease onset, years (mean ± SD)	34.6 ± 10.0	41.7 ± 13.7	31.0 ± 18.2	//	*p* = 0.032^a^
Disease duration, months (mean ± SD)	10.9 ± 7.0	8.2 ± 7.7	5.5 ± 5.6	//	*p* = 0.014^a^
Volume of brain white matter lesions, mm^3^ (mean ± SD)	9507.57 ± 8823.45	2843.68 ± 4986.48	10,493.41 ± 19,640.88	//	*p* = 0.039^a^
ON, prevalence (%)	11 / 28 (39.3%)	15 / 24 (62.5%)	22 / 28 (78.6%)	//	*p* = 0.011^b^
Bilateral ON, prevalence in patients with ON (%)	1 / 11 (9.1%)	6 / 15 (40.0%)	14 / 22 (63.6%)	//	*p* = 0.011^b^
Relapsing ON, prevalence in patients (%)	0 / 11 (0.0%)	5 / 15 (33.3%)	14 / 22 (63.6%)	//	*p* = 0.002^b^
ON number (mean ± SD)	0.43 ± 0.57	1.13 ± 1.19	2.93 ± 2.98	//	*p* < 0.001^a^
Time from first ON, months (mean ± SD)	89.6 ± 70.2	107.8 ± 87.4	73.8 ± 72.2	//	*p* = 0.456^a^
Time from last ON, months (mean ± SD)	68.6 ± 51.0	72.0 ± 68.2	29.8 ± 35.9	//	*p* = 0.047^a^

RRMS: relapsing-remitting multiple sclerosis; AQP4 + NMOSD: aquaporin 4-antibody neuromyelitis optica spectrum disorder; MOGAD: myelin oligodendrocyte glycoprotein antibody–associated disease; HCs: healthy controls.

a *p*-value using ANOVA (Analysis of Variance) test to compare the groups.

b Chi-square test for independence to compare the groups.

**Table 2.** Clinical characteristics of the population of study.

Demographic characteristics	ON + IDD (*n* = 48)	ON− IDD (*n* = 32)	HCs (*n* = 32)	*p*
Gender, M / F (male %)	13 / 32 (40.6%)	12 / 48 (25.0%)	10 / 22 (31.3%)	*p* = 0.336^b^
Age, years (mean ± SD)	42.2 ± 16.0	45.7 ± 13.4	35.2 ± 12.4	*p* = 0.015^a^
Age at disease onset, years (mean ± SD)	33.6 ± 15.9	38.3 ± 13.0	//	*p* = 0.074^a^
Disease duration, months (mean ± SD)	8.7 ± 7.3	7.5 ± 6.8	//	*p* = 0.448^a^
Volume of brain white matter lesions, mm^3^ (mean ± SD)	6885.86 ± 11,710.45	7159.35 ± 7792.89	//	*p* = 0.920^a^

ON+ IDD: patients with inflammatory demyelinating diseases and at least a previous optic neuritis episode; ON− IDD: patients with inflammatory demyelinating diseases without history of previous optic neuritis episode; HCs: healthy controls.

a *p*-value using ANOVA (Analysis of Variance) test to compare the groups.

b Chi-square test for independence to compare the group.

IDD patients had average disease duration of 8.2 ± 7.1 years. Disease duration did not differ significantly between ON+ IDD and ON− IDD. Forty-eight patients (60%) experienced at least one previous ON, and among them, ON affected both eyes in 21/48 (43.75%) and was relapsing in 21/48 (39.58%). The average number of inflammatory events was 2.52 ± 2.32, with 22/48 (45.83%) patients reporting a single episode, 10/48 (20.83%) having experienced two previous ON and 16/48 (33.33%) reporting three or more previous ON. The average time-gap from the first ON was 87.5 ± 76.2 months, while the time-gap from the last one was 51.1 ± 52.5 months.

Clinical characteristics of the different disease groups are summarised in Table 1. The prevalence of previous ON and the mean number of ON were higher in MOGAD and AQP4 + NMOSD when compared with RRMS (both *p* < 0.001). The same groups reported higher percentages of bilateral ON (*p* = 0.011) and relapsing ON episodes (*p* = 0.002).

### Effect of previous ON on OC MTR in IDD

**Figure 2.**
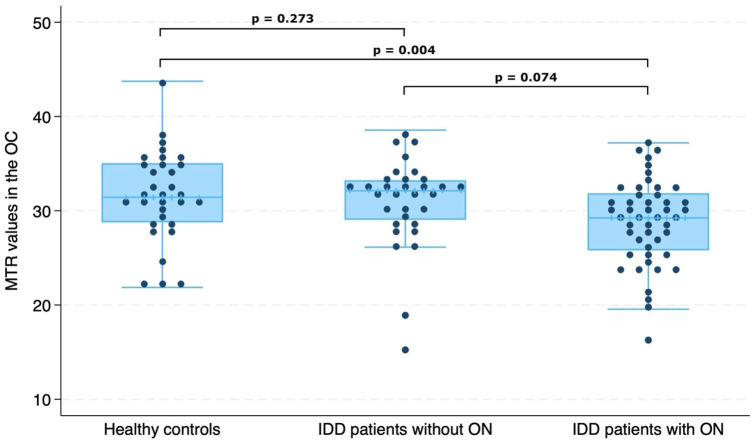
Comparison of magnetisation transfer ratio (MTR) in the optic chiasm (OC) between patients with inflammatory demyelinating diseases (IDD) without previous optic neuritis (ON) (*n* = 32), patients with previous ON (*n* = 48) and healthy controls (*n* = 32).

Chiasmal MTR values in ON− and ON+ patients are reported in Table 3. ON+ IDD patients showed lower MTR values compared with HCs (regression coefficient (RC) = −3.33, 95% confidence interval (CI) = −5.56 to −1.111, *p* = 0.004) and slightly lower MTR values compared with ON− IDD patients although non-significant (Figure 2). OC MTR values were not significantly different between HCs and ON− IDD patients.

**Table 3.** Chiasmal MTR values in the different diseases according to the number of previous optic neuritis (ON).

Subgroup	IDD (*n* = 81)	RRMS (*n* = 28)	AQP4 + NMOSD (*n* = 24)	MOGAD (*n* = 28)	HCs (*n* = 32)
Subjects without ON	(*n* = 32) 30.99 ± 4.76	(*n* = 17) 32.90 ± 2.59	(*n* = 9) 28.37 ± 7.27	(*n* = 6) 29.49 ± 2.65	(*n* = 32) 31.65 ± 4.93
Subjects with previous ON	(*n* = 48) 28.87 ± 4.58	(*n* = 11) 29.60 ± 4.58	(*n* = 15) 28.55 ± 4.18	(*n* = 22) 28.73 ± 4.99	//
Subjects with 1 ON	(*n* = 22) 29.80 ± 4.08	(*n* = 10) 29.64 ± 4.83	(*n* = 8) 30.25 ± 4.10	(*n* = 4) 29.31 ± 2.57	//
Subjects with 2 ON	(*n* = 10) 28.75 ± 2.39	(*n* = 1) 29.19 ± 0.00	(*n* = 3)27.52 ± 4.23	(*n* = 6) 29.29 ± 1.35	//
Subjects with ⩾ 3 ON	(*n* = 16) 27.68 ± 6.02	(*n* = 0)	(*n* = 4) 25.93 ± 3.54	(*n* = 12) 28.26 ± 6.67	//

IDD: inflammatory demyelinating diseases; RRMS: relapsing-remitting multiple sclerosis; AQP4 + NMOSD: aquaporin 4-antibody neuromyelitis optica spectrum disorder; MOGAD: myelin oligodendrocyte glycoprotein antibody–associated disease; HCs: healthy controls.

**Figure 3.**
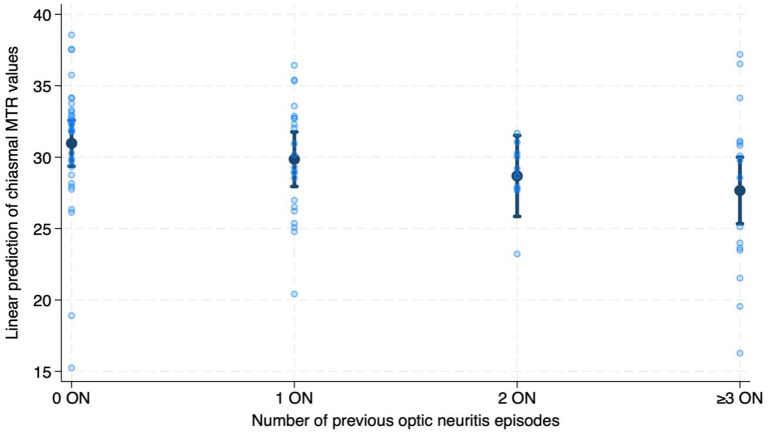
Association between magnetisation transfer ratio (MTR) values and number of previous optic neuritis (ON) episodes. Predicted chiasmal MTR values from number of previous optic neuritis from the linear regression model adjusted for age, sex and MRI upgrade.

**Figure 4.**
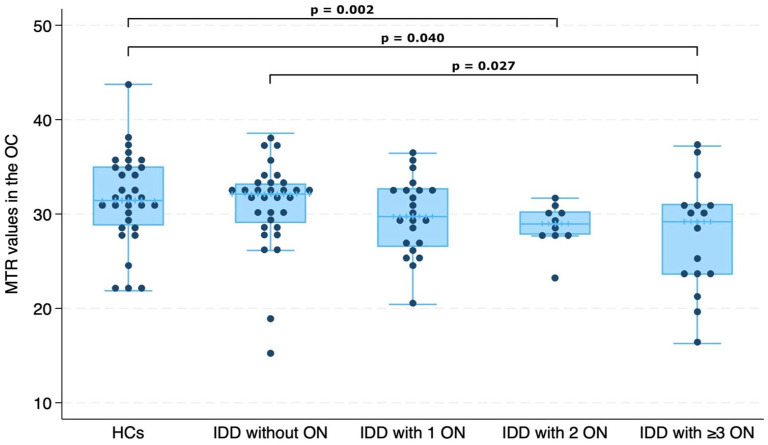
Comparison between magnetisation transfer ratio (MTR) values in the optic chiasm (OC) and number of previous optic neuritis (ON) episodes in inflammatory demyelinating diseases (IDD).

Higher numbers of previous ON episodes were associated with lower chiasmal MTR (RC = −1.15, 95% CI = −1.819 to −0.490, *p* = 0.001) (Figure 3). We observed that IDD patients with two previous ON and those with at least three previous ON episodes had lower chiasmal MTR values than HCs (*two ON vs HCs*: RC = −3.58, 95% CI = −6.892 to −0.170, *p* = 0.040; *three or more ON vs HCs*: RC = −4.62, 95% CI = −7.558 to −1.674, *p* = 0.002) (Figure 4). Patients with three or more previous ON episodes also showed lower MTR values when compared with ON− patients (RC = −3.38, 95% CI = −6.374 to −0.384, *p* = 0.027) (Figure 4).

### Differences in OC MTR between RRMS, AQP4 + NMOSD, MOGAD and HCs

ON+ RRMS patients had lower MTR values than ON− RRMS patients (RC = −2.994, 95% CI = −5.812 to −0.177, *p* = 0.038), while no significant difference was observed comparing ON+ vs ON− AQP4-NMOSD and ON+ vs ON− MOGAD.

**Figure 5.**
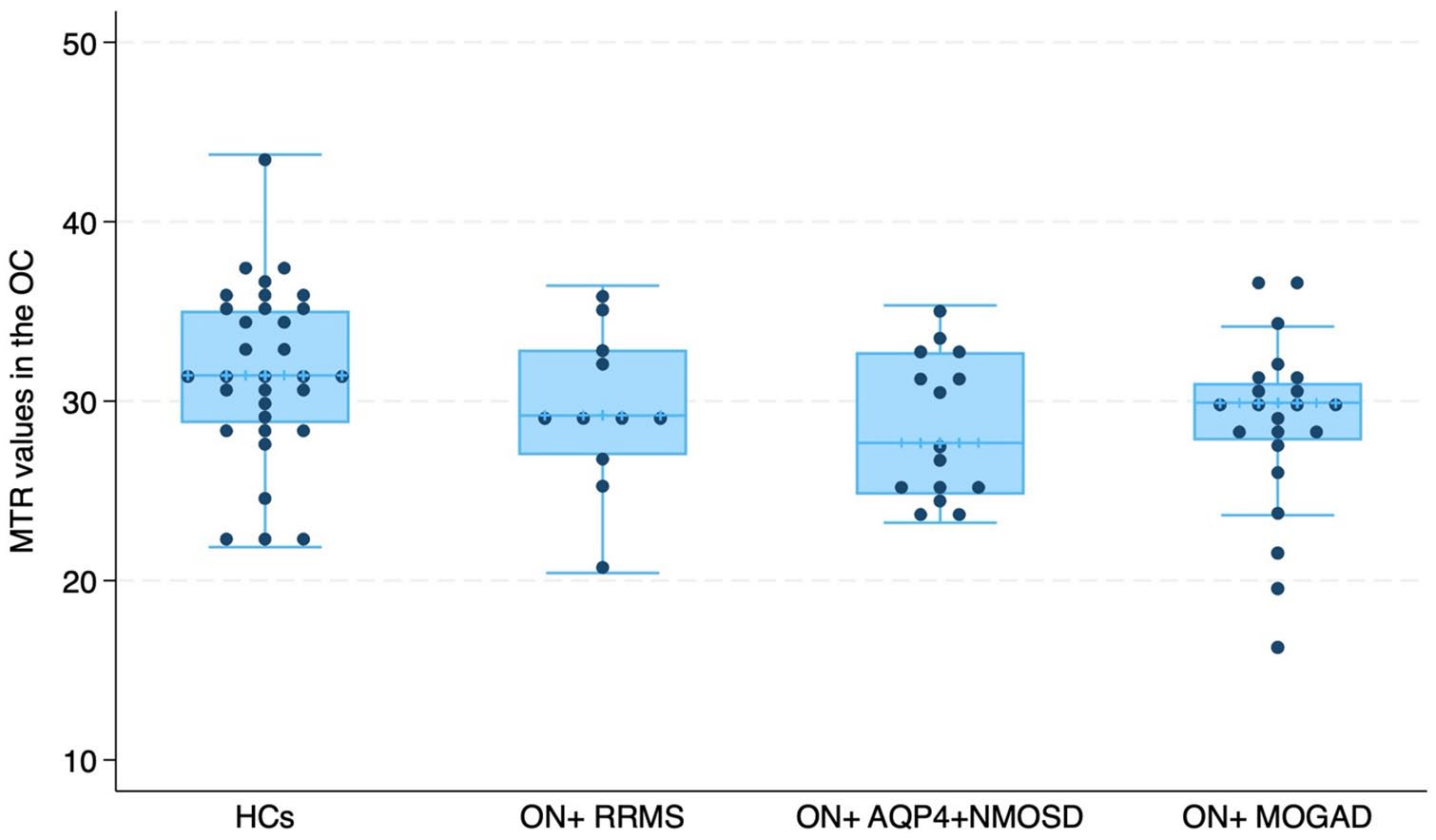
Comparison of magnetisation transfer ratio (MTR) in the optic chiasm (OC) between relapsing-remitting multiple sclerosis (RRMS) patients (*n* = 11), aquaporin 4-antibody neuromyelitis optica spectrum disorder (AQP4 + NMOSD) (*n* = 15) and myelin oligodendrocyte glycoprotein antibody–associated disease (MOGAD) (*n* = 22) with previous optic neuritis (ON) and healthy controls (HCs) (*n* = 32).

For ON+ patients, AQP4 + NMOSD and MOGAD demonstrated lower OC MTR than HCs (respectively: RC = −3.547, 95% CI = −6.527 to −0.567, *p* = 0.020; RC = −3.900, 95% CI = −6.707 to −1.092, *p* = 0.007), while no differences were found between RRMS and HCs (Figure 5).

In AQP4 + NMOSD, ON− patients had lower chiasmal MTR than HCs (RC = −3.870, 95% CI = −7.468 to −0.272, *p* = 0.035), while no difference was found comparing ON− RRMS and ON− MOGAD patients with HCs.

Combining both ON+ and ON− patients and adjusting for the number of previous ON episodes, we observed that AQP4 + NMOSD and MOGAD exhibited lower OC MTR values than HCs (respectively: RC = −3.215, 95% CI = −5.998 to −0.432, *p* = 0.024; RC = −2.938, 95% CI = −5.850 to −0.026, *p* = 0.048). However, no significant differences were found between RRMS and HCs. Furthermore, our results indicated that AQP4 + NMOSD had lower MTR values compared to RRMS (RC = −2.923, 95% CI = −5.483 to −0.363, *p* = 0.026), while the difference between MOGAD and RRMS was non-significant.

### Associations between OC MTR and OCT pRNFL

Both ON+ and ON− IDD patients had lower pRNFL thicknesses than HCs (*ON* *+* *IDD vs HCs*: 74.03 μm ± 20.75 vs 102.07 μm ± 9.95, RC = −28.584, 95% CI = −38.257 to −18.910, *p* < 0.001; *ON− IDD vs HCs*: 92.76 μm ± 8.47 vs 102.07 μm ± 9.95, RC = −11.411, 95% CI = −21.592 to −1.231, *p* = 0.029). We also found that ON + IDD had lower pRNFL thickness compared with ON− IDD (*average pRNFL*: 74.03 μm ± 20.75 vs 92.75 μm ± 8.47, RC = −17.7172, 95% CI = −25.248 to −9.096, *p* < 0.001).

**Figure 6.**
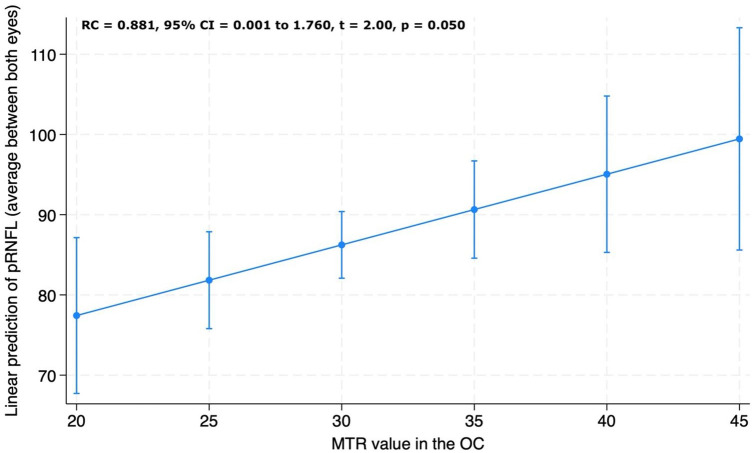
Association between peripapillary retinal nerve fibre layer (pRNFL) thickness and magnetisation transfer ratio (MTR) values in the optic chiasm (OC). Visual acuity is reported as average pRNFL thickness between the two eyes. Adjusted predictions of visual acuity from chiasmal MTR values from the linear regression model adjusted for age, sex and MRI upgrade.

For the overall IDD group, lower chiasmal MTR was associated with lower pRNFL thickness measured both as (1) average pRNFL between the two eyes (RC = 0.881, 95% CI = 0.001 to 1.760, *p* = 0.050) (Figure 6) and (2) thinner pRNFL (RC = 1.129, 95% CI = 0.199 to 2.059, *p* = 0.018).

### Associations between OC MTR and OCT macular GCIPL

ON+IDD patients had lower macular GCIPL thicknesses than HCs (71.33 μm ± 15.77 vs 94.18 μm ± 11.12, RC = −22.873, 95% CI = −32.769 to −12.977, *p* < 0.001) and ON− IDD (71.33 μm ± 15.77 vs 90.82 μm ± 4.67, RC = −19.027, 95% CI = −27.354 to −10.699, *p* < 0.001), while we observed no differences between ON− IDD and HCs.

No significant associations were detected between GCIPL and OC MTR for the whole IDD group (RC = 0.554, 95% CI = −0.371 to 1.480, *p* = 0.235) nor within each disease group.

### Associations between OC MTR and visual acuity

LogMAR acuity was worse in ON+ IDD than in ON− IDD patients (0.32 ± 0.47 vs 0.13 ± 0.19; RC = −0.207, 95% CI = −0.352 to −0.062, *p* = 0.005).

**Figure 7.**
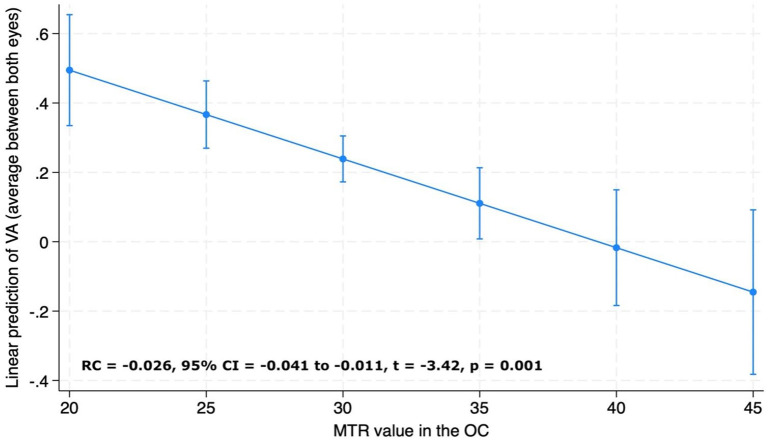
Association between visual acuity (VA) and magnetisation transfer ratio (MTR) values in the optic chiasm (OC). VA is reported as average visual acuity between the two eyes. Adjusted predictions of visual acuity from chiasmal MTR values from the linear regression model adjusted for age, sex and MRI upgrade.

For the whole IDD group, lower chiasmal MTR was associated with lower logMAR acuity measured both as (1) average visual acuity between the two eyes (RC = −0.026, 95% CI = −0.041 to −0.011, *p* = 0.001) (Figure 7) and (2) visual acuity of the more affected eye (RC = −0.037, 95% CI = −0.059 to −0.015, *p* = 0.001).

Finally, we found that in AQP4 + NMOSD, there was a strong association between the visual acuity and MTR measures (RC = −0.036, 95% CI = −0.068 to −0.003, *p* = 0.032), while for MOGAD, the associations were not significant (RC = −0.003, 95% CI = −0.028 to 0.021, *p* = 0.795).

## Discussion

This study provides insights into the mechanisms of OC damage in IDD with and without previous ON. ON+ IDD patients showed lower MTR values compared with HCs and ON− IDD patients, with a higher number of previous ON episodes associated with lower OC MTR. In our study population, chiasmal involvement was more pronounced in patients with AQP4 + NMOSD and MOGAD than in those with MS. In addition, lower OC MTR values correlated with reduced pRNFL thickness and poorer visual outcomes.

The strong association between the number of previous ON episodes and chiasmal MTR aligns with previous research on post-acute changes following inflammatory optic nerve lesions.^17,26^ Lower MTR values are likely to reflect reduced microstructural integrity^10^ which can result from both demyelination and inflammatory-related changes during the acute phase of ON,^13,27^ while correlate with demyelination, remyelination and neuroaxonal loss in the long-term,^28–30^ representing a useful marker of chronic damage and repair.^13^

In our study, we observed that OC MTR values were lower in ON+ IDD patients, compared with HCs. We also noted weak evidence for lower OC MTR in ON+ IDD compared with ON− patients. A previous study by Juenger et al.^19^ investigated the role of chiasmal measures as an imaging marker for anterior optic pathway damage. The authors demonstrated significant group differences between NMOSD patients and HCs, and strong associations of OC measures with structural and clinical factors, indicating that OC dimensions could discriminate patients with ON from controls. Our results align with those previously reported confirming that OC metrics are strongly associated with the number of previous ON episodes. The process of visual recovery following an inflammatory episode may involve several factors, including the resolution of acute inflammatory processes, the accumulation of new sodium channels in demyelinated axons and the plasticity of central synaptic networks. These intricate mechanisms may interact with the microstructural integrity of the OC, as reflected by MTR values, and play a role in determining the extent of visual restoration or impairment in individuals with IDD and a history of ON.^16^ However, these processes may be incomplete and the occurrence of new acute ON episodes could progressively exhaust the mechanisms responsible for recovery. Therefore, we hypothesise that the greater reduction in OC MTR following multiple ON episodes could result from two related factors: direct axonal transection and degeneration caused by multiple inflammatory attacks and/or a progressive impairment of the post-inflammatory recovery processes due to the accumulation of damage associated with relapsing ON. This hypothesis gains support from the correlation we observed between OC MTR values and pRNFL thickness, a well-recognised marker of neuroaxonal loss in the anterior visual pathways. pRNFL has been extensively used to investigate the optic nerve in IDD, showing robust associations with brain atrophy, neurophysiological measures and clinical outcomes.^31–33^ The association we observed between OC MTR values and pRNFL in our study underscores the role of MTR as a measure of chronic biological damage in the anterior optic pathway. Interestingly, we did not observe significant relationships between GCIPL and chiasmal metrics. However, GCIPL data were available for only 49 out of 80 patients. It is likely, therefore, that this analysis was underpowered to be able to establish significance, possibly as a consequence of high standard errors even though the association was in a physiologically plausible direction (RC = 0.554, 95% CI = −0.371 to 1.480, *p* = 0.235).

Our study also revealed an association between lower OC MTR values and poorer visual acuity. The OC is recognised as a region with high axonal density, and its damage has been linked to a decline in visual function.^19^ Our results suggest that microstructural damage occurring in both the optic nerve and OC following ON indicates a broader pattern of microstructural damage. This damage appears to contribute to a reduction in overall visual function, in line with previous studies.^9,19^ The linear association we observed between OC MTR measures and visual acuity further emphasises the role of chiasmal MTR as a useful measure for assessing both biological and clinical damage in the anterior visual pathway.

Of note, we observed that in MOGAD, there was no association between visual acuity and MTR measures. This finding is consistent with the observation that, despite significant microstructural damage of the anterior visual pathway, MOGAD patients exhibit better functional recovery compared with AQP4 + NMOSD.^7,8^ Further studies will be necessary to better understand the mechanisms underlying this function–structure discordance.

The involvement of the OC in ON lesions has been well documented in cases of AQP4 + NMOSD, but it is less common in MS and MOGAD.^2,15^ Despite these differences, in our population we observed that ON+ AQP4 + NMOSD and MOGAD had lower MTR values when compared to HCs. In contrast, ON+ RRMS showed MTR values that were non-significantly lower than those of the control group. These findings align with recent research which challenges the conventional assumption that the frequency of OC involvement in AQP4 + NMOSD and MOGAD significantly differs.^15^ While the percentage of OC involvement in these two antibody-mediated diseases is not dissimilar (20% in AQP4 + NMOSD vs 16% in MOGAD), Tajfirouz et al.^15^ noted that the characteristics of OC damage seem to vary between these conditions. Specifically, the authors proposed that OC involvement in MOGAD could be attributed to an ‘extension’ of long anterior lesions, whereas in AQP4 + NMOSD, the damage may be more directly associated with the posterior localisation of the lesion.^15^ Interestingly, in both disorders, the damage appears to be secondary to an inflammatory process within the optic nerve. Our findings support this hypothesis, by revealing that the higher number of ON episodes recorded in MOGAD and AQP4 + NMOSD patients (with a mean of 2.93 and 1.13, respectively) resulted in more severe damage of the OC, as indicated by lower MTR values. However, we also observed that in MOGAD and AQP4 + NMOSD patients, OC MTR values remained lower than those of HCs, even after accounting for the number of previous ON episodes. This observation might suggest (1) the existence of damage independent from relapse which could subtly contribute to the decline in microstructural integrity within the OC and/or (2) the occurrence of subclinical ON. This phenomenon is notably pronounced in AQP4 + NMOSD, who reported lower MTR values than those of RRMS patients. In addition, in this group, ON− patients showed lower MTR values compared with HCs. Juenger et al.^19^ have previously reported a reduction in chiasmal dimensions in ON− NMOSD, suggesting that microstructural changes could occur in the optic pathway of NMOSD patients independent of previous ON. Our results corroborate this hypothesis, underscoring the complex relationship between ON, OC involvement and the distinct characteristics of IDD subtypes and emphasising the importance of considering these factors when assessing the impact of these diseases on visual function and structural integrity.

Our study has limitations: first, it adopts a cross-sectional design, and we acknowledge that a longitudinal, prospective cohort approach would better assess damage and repair processes during the post-acute phase. Second, the absence of images acquired for the evaluation of optic nerve lesions during the acute phase limited our ability to assess the influence of these lesions on chronic optic nerve damage. However, we addressed this second limitation by building robust statistical models and creating associations based on the available data, including OCT and visual data and volume of brain white matter lesions. Finally, we recognise that the OC is a small structure, as is the optic nerve, making investigation of the anterior visual pathway challenging. Nonetheless, compared with the optic nerve, the OC is less susceptible to motion artefact, less variable in morphology and possesses larger in dimensions, reinforcing its viability as a target for quantitative MRI. In addition, the fact that our results, demonstrate significant associations between previous optic neuritis and OC damage, along with strong associations between OCT and clinical data, support the proposition that this structure could play an important role as an accessible target for assessing the visual pathway in inflammatory diseases.

In conclusion, our findings suggest that OC damage in ON, as demonstrated by MTR, is linked to the number of inflammatory ON episodes experienced by IDD patients and reflects pathological changes occurring in the anterior visual pathways within IDD. As optic nerve MRI remains challenging due to its small dimensions, mobility and susceptibility to artefacts, MTR OC imaging could provide additional information for evaluating the anterior visual pathways in IDDs.
